# Dendritic Cells in HIV/SIV Prophylactic and Therapeutic Vaccination

**DOI:** 10.3390/v12010024

**Published:** 2019-12-24

**Authors:** Eun-Ju Ko, Marjorie Robert-Guroff

**Affiliations:** 1 College of Veterinary Medicine, Jeju National University, Jeju 63243, Korea; 2 Interdisciplinary Graduate Program in Advanced Convergence Technology & Science, Jeju National University, Jeju 63243, Korea; 3 Vaccine Branch, National Cancer Institute, National Institutes of Health, Bethesda, MD 20892, USA

**Keywords:** dendritic cells, human/simian immunodeficiency virus, vaccine, antigen-specific responses

## Abstract

Dendritic cells (DCs) are involved in human and simian immunodeficiency virus (HIV and SIV) pathogenesis but also play a critical role in orchestrating innate and adaptive vaccine-specific immune responses. Effective HIV/SIV vaccines require strong antigen-specific CD4 T cell responses, cytotoxic activity of CD8 T cells, and neutralizing/non-neutralizing antibody production at mucosal and systemic sites. To develop a protective HIV/SIV vaccine, vaccine regimens including DCs themselves, protein, DNA, mRNA, virus vectors, and various combinations have been evaluated in different animal and human models. Recent studies have shown that DCs enhanced prophylactic HIV/SIV vaccine efficacy by producing pro-inflammatory cytokines, improving T cell responses, and recruiting effector cells to target tissues. DCs are also targets for therapeutic HIV/SIV vaccines due to their ability to reverse latency, present antigen, and augment T and B cell immunity. Here, we review the complex interactions of DCs over the course of HIV/SIV prophylactic and therapeutic immunizations, providing new insights into development of advanced DC-targeted HIV/SIV vaccines.

## 1. General Characteristics of Dendritic Cells (DCs)

Dendritic cells (DCs) play a central role in orchestrating both innate and adaptive immune responses. They are found in an immature state in most peripheral tissues, including skin and respiratory and intestinal mucosa, as well as in blood. As innate immune cells, immature DCs in peripheral tissues can recognize pathogens by their surface pattern recognition receptors (PRRs) such as toll-like receptors (TLRs) and mannose receptors. They can also produce pro-inflammatory cytokines such as interleukin (IL)-6, IL-12, and tumor necrosis factor (TNF)-alpha to initiate inflammation at the site of infection and remove pathogens by phagocytosis. Upon antigen uptake, they become the most potent antigen-presenting cells (APCs) and migrate to secondary lymphoid tissues to initiate adaptive immune responses. During the migration, DCs process pathogens into antigenic peptides and increase expression of their activation markers such as CD40, CD80, CD86, and major histocompatibility complex (MHC) class II molecules for enhancing antigen presentation to naïve CD4 T cells [1,2].

Antigen presentation by matured DCs is required to initiate antigen-specific CD4 T cell responses in lymph nodes. The DC–CD4 T cell interactions between MHC class II molecules and T cell receptors induce T helper (Th) 1, Th2, Th17, or regulatory T cell responses dependent on the pathogen encountered, the cytokine/chemokine levels in the microenvironment, and the type of PRRs activated on the DCs. The antigenic peptides presented on MHC class I molecules of DCs can activate cytotoxic CD8 T cells as well. The antigen-specific CD4 T helper cells activated by DCs can interact with antigen-sensitized B cells and induce isotype class switching, somatic hypermutation, and development of memory and plasma cells in germinal centers [1]. DCs also are involved in B cell activation by transferring retained antigen to naïve B cells and giving cell-bound signals to B cells for class switching [3]. Taken together, the DC population is critical for both innate and adaptive immune responses against pathogen invasion.

Human DCs are broadly divided into CD11c-expressing myeloid DCs (mDCs), also known as conventional DCs (cDCs), and CD123-expressing plasmacytoid DCs (pDCs). A specialized subset of mDCs expressing CD207 (langerin) is present in epidermal tissue and called Langerhans cells (LCs). Generally, mDCs have high phagocytic capacity in the immature state and produce pro-inflammatory cytokines to eliminate invading pathogens and initiate inflammation in local areas. To initiate the inflammatory responses, mDCs express various PRRs such as TLR on their surface. Human pDCs exhibit plasma cell morphology and express BDCA (blood DC antigen)-2 and BDCA-4 in addition to CD123 while mDCs present BCDA-1 and BCDA-3. Expression of TLR7 and 9 on pDCs within endosomal compartments allow them to recognize viral nucleic acids effectively. Upon activation of the TLR7 and 9 signaling pathway by viral infection, pDCs produce a large amount of type 1 interferon (IFN) with antiviral activity. Both mDCs and pDCs exhibit anti-viral capacity by secretion of cytokines, antigen presentation, and T cell activation [1,4].

## 2. Dual Roles of DCs in HIV Infection

As summarized above, DCs provide critical antiviral activities; however, they can also facilitate viral infection. Human immunodeficiency virus (HIV) and simian immunodeficiency virus (SIV) infections induce a severe immune-deficient condition due to a decreased number of CD4 T cells [5]. HIV/SIV infects CD4 T cells mainly by targeting CD4 and the chemokine CC receptor 5 (CCR5) but can also infect DCs through a number of receptors including CCR5, chemokine CXC receptor 4 (CXCR4), and the DC-specific intercellular adhesion molecule-3-grabbing nonintegrin (DC-SIGN) on the surface of DCs, which allows HIV and SIV envelope binding and attachment. In addition to this glycoprotein-dependent viral capture by DCs, envelope independent lipid-dependent viral capture has been described [6,7]. In fact, some studies have shown that epidermal LCs and DCs are the main viral target cells rather than CD4 T cells in early SIV infection by the vaginal route [8,9,10]. Other studies have revealed that HIV infection induces DC activation and maturation by various mechanisms including TLR pathways, stimulator of interferon genes (STING), and the cyclic guanosine monophosphate-adenosine monophosphate synthase (cGAS) pathway and a mitochondrial antiviral-signaling protein (MAVS)-dependent pathway [11,12,13,14,15]. However, even though HIV/SIV uptake enhances DC maturation and migration to secondary lymphoid organs, viral replication is less efficient in DCs than in CD4 T cells [4]. Based on which receptor/binding pathway is used for HIV infection, the virus is either transmitted to CD4 T cells or degraded without transmission. When the virus binds to langerin on LCs, the virion is degraded rapidly in acidic endosomal compartments [16]. In contrast, when binding to DC-SIGN occurs, the virion can be delivered to uninfected T cells without complete degradation [17]. In chronic HIV infection, circulating memory T cells are the major HIV reservoir (for reviews, see [18,19,20]). It has been suggested that, in a lymphoid organ, DCs may activate the resting memory T cells and cause reactivation of HIV transmission [4]. A recent study in chronically HIV-infected individuals showed that CD8 T cells not only responded to adapted (mutated) HIV epitopes, resulting in killing of CD4 T cells, but also simultaneously led to greater dendritic cell maturation, trans-infection of CD4 T cells, and overall persistence of the mutated viral strains, providing HIV an advantage [21]. In addition, HIV itself also induces suppression of DC maturation and impairs DC functions such as cytokine production [22,23]. Taken together, DCs play dual roles in viral spread and viral elimination after HIV infection.

## 3. HIV Prophylactic Vaccine Development and DCs

Vaccination is the most effective way to provide protection against infection by establishing strong memory immune responses in advance of pathogen exposure. Most HIV and SIV vaccine studies have focused on antigen-specific T and B cell responses. HIV vaccines are required to induce strong and durable CD8 T cells and neutralizing and non-neutralizing antibody responses to provide effective protection or at least delay HIV infection [24,25]. However, to date, only one of six clinical HIV vaccine trials, the RV144 trial in Thailand, has shown any protective efficacy, and that was a modest 31.2% (for reviews, see [26,27,28,29]). To improve vaccine efficacy, many studies have reported that DCs play a critical role as a cellular adjuvant in HIV vaccine mechanisms by improving cell-mediated responses, antibody production, and T cell memory following HIV vaccination [2,30,31,32]. As described above, DCs regulate antigen-specific immune responses after infection. Similarly, DCs capture vaccine antigens at the site of immunization, process them into peptides during migration and maturation, and present them to naïve T cells in secondary lymphoid tissues to elicit antigen-specific T and B cell responses. Therefore, DC functions directly affect vaccine efficacy. Many studies have investigated the role of DCs in immune mechanisms of HIV vaccine protection, and various prophylactic HIV vaccine platforms have tried to induce DC activation to increase vaccine efficacy. Here, we review studies using DCs as components of HIV vaccines, including recent attempts to harness the power of these critical APCs for improved protective efficacy.

Some of the earliest attempts to use DCs as HIV vaccines mimicked studies on immunotherapy of cancer in which peptide-pulsed autologous DCs were used for immunization and induced peptide-specific cytotoxic T lymphocytes (CTLs) with therapeutic benefit [33]. In most cases, DCs for immunotherapeutic use were derived from blood monocytes by treatment with granulocyte-macrophage colony stimulating factor (GM-CSF) and IL-4. These monocyte-derived DCs (MoDCs) have the capacity to secrete pro-inflammatory cytokines such as IL-12p70 and TNF-α and to capture and process antigens to initiate adaptive immune responses [34,35]. For example, rhesus macaques intravenously immunized with autologous MoDCs pulsed with a cocktail of HIV peptides developed cell mediated immunity that was enhanced compared to immunization with peptides in incomplete Freund’s adjuvant and associated with reduced viremia and lessened mortality following a SHIV_89.6P_ challenge [36]. Peptide loaded DCs from blood monocytes, LCs, and interstitial, dermal-intestinal DCs derived from stem cells of neonatal cord blood were shown to induce HIV-specific polyfunctional CD8 T cell responses from naïve CD8 T cells of HIV-negative humans and neonates after in vitro DC-T cell co-culture [37]. MoDCs from non-human primates pulsed with inactivated SIV in vitro were shown to elicit cellular and humoral immune responses following re-infusion in vivo [38]. These types of studies demonstrated the potential of DC-based HIV vaccines. However, the MoDCs were generated in vitro; therefore, the overall process was relatively laborious. The approach was soon followed by direct immunization with protein antigens or DNA components, which was shown to directly transfect DCs at the immunization site [39,40]. Both protein antigens and DNA involved DCs as intermediaries of immune responses. Further improvements to the DC strategy involved targeting of immunogens to the cells using a variety of mechanisms to facilitate their capture and presentation.

A common method of DC targeting has been to conjugate antigen to monoclonal antibodies specific for DC endocytic receptors such as DEC-205 and Clec9A [1]. This approach requires administration with an additional adjuvant to mature the DCs, in the absence of which tolerance ensues. Using such an approach, HIV p24 conjugated to DEC-205 monoclonal antibody was shown to be strongly immunogenic in mice, rhesus macaques, and humans, eliciting both T and B-cell responses [41]. DNA can also be targeted to DC receptors. A DNA vaccine encoding a fusion protein of HIV gag with a DEC-205 single chain Fv antibody induced better antigen presentation efficacy, higher antibody levels, and increased functionally activated T cell responses [42]. Moreover, administration of a Gag protein vaccine targeting DEC-205 before boosting with the fusion DNA elicited better protection against recombinant gag-expressing vaccinia virus by inducing rapid and improved CD8 T cell responses [30].

All HIV antigens have been targeted for inducing cellular immunity, but the envelope protein has been of special interest as the target of neutralizing antibodies. Moreover, vaccine-induced non-neutralizing antibodies against the V1/V2 region of HIV gp120 were shown to be associated with protection in the RV144 clinical trial [25,43,44]. Many vaccine strategies, in addition to targeting to endocytic receptors, have tried to increase anti-envelope immune responses and hence HIV vaccine efficacy by enhancing DC activation and maturation. One novel strategy replaced sialic acid on the carbohydrate chains of HIV gp120 with Galα1-3Galβ1-4GlcNAc-R epitopes, ligands for abundantly naturally expressed anti-Gal antibody [45]. The rationale was to allow in vivo immune complex formation between the gp120 (alphagal) with anti-Gal, leading to interaction between the antibody Fc component and the Fcγ receptors on APC, including DCs. Immunization of mice with this construct compared to mice that received unmodified gp120 showed increased immunogenicity, inducing 100-fold higher antibody titers with better neutralizing activity and enhanced antigen-specific T cell responses [45]. Another novel approach made use of chimeric HIV-1 gp140 trimer fused with CD40 ligand (CD40L) to directly target DCs [46]. The CD40L bound to CD40 on DCs resulted in signaling and induction of DC maturation. The plasmid encoding chemokine macrophage inflammatory protein-1 alpha (MIP-1α) has been shown to recruit immature DCs to the site of DNA inoculation in mice, while fms-like tyrosine kinase 3 ligand (Flt3L), a potent DC-specific growth factor, has expanded and matured DCs in both mice and humans. An HIV Env DNA vaccine with plasmids encoding MIP-1α and Flt3L exhibited DC recruitment at the site of immunization and induced CD11b, CD80, CD83, and MHC class II, the activation and the maturation markers of DCs. This vaccination strategy achieved better protection against challenge with recombinant HIV Env-expressing vaccinia virus in the mouse model compared to the DNA vaccine alone or with either MIP-1α or Flt3L only [40]. It was previously shown that a DNA vaccine encoding HIV-1 gp120 linked to the extracellular domain of Flt3L induced DC expansion, higher frequencies of gp120-specific CD8 T cells, and higher anti-gp120 antibody production. The antigen-specific CD8 T cell responses were durable and were maintained for 16 weeks after the boost immunization [47]. Additional molecules have also been used, including cytotoxic T lymphocyte antigen 4 (CTLA4), which binds to APCs, and programmed cell death protein (PD)-1, as both PD-L1 and PD-L2 are expressed on DCs. Depending on the route of immunization, a DNA vaccine of HIV gp120 fused with CLTA4 induced 16-fold higher antibody titers compared to DNA encoding gp120 only, while cellular responses were increased up to eight-fold [48]. A DNA vaccine composed of HIV Gag fused to PD-1 was shown to elicit polyfunctional and long-lived CD8 T cell activities by enhancing IL-12 secretion and engaging the antigen cross-presentation pathway of DCs [49].

With the advent of nanobiology, additional new approaches for enhancing uptake and antigen presentation by DCs have emerged (for reviews, see [50,51]). For example, HIV gag p24 protein coated onto polylactic acid colloidal biodegradable nanoparticles could elicit ex vivo activation of MoDCs from HIV infected individuals and induce gag-specific cytotoxic cellular immune responses, CD4 and CD8 T cell proliferation, and cytokine secretion [52]. Similarly, gold nanostructures can facilitate antigen loading and DC targeting, thus promoting cellular and humoral immunity [53]. A recent ex vivo study evaluated gold nanoparticles bearing high-mannoside-type oligosaccharides coupled to HIV p17 peptides and found increased HIV-specific CD4 and CD8 T cell proliferation and cytokine secretion compared to use of peptides alone [54]. The high mannose ligands provided an adjuvant effect, as these particles target C-type lectins such as DC-SIGN present on DCs as well as macrophages. A somewhat similar strategy was previously used to target DCs in the skin. DNA expressing all HIV proteins except integrase and coupled to mannosylated particles (termed DermaVir) was developed as a topical vaccine. Following application to the skin, the targeted and the transduced DCs migrated to underlying lymph nodes as potent APCs and elicited virus-specific CD4 and CD8 memory T cells [55]. With regard to elicitation of functional anti-Env responses, a recent study illustrated the potential of a nanoparticle approach. Although the conformation of the viral Env is critical for elicitation of antibodies with broadly neutralizing activity, a minimal epitope approach was utilized to focus responses on a specific neutralizing site [56]. Star dendrimers conjugated with an HIV V3 peptide, a universal CD4^+^ T cell helper peptide, and/or a TLR7/8 agonist as adjuvant were shown to be taken up by DCs and to elicit high titer V3 specific antibodies. Neutralizing antibodies were not induced, however, even following boosting with HIV Env trimers, perhaps due to the angle at which the induced antibody approached a native Env epitope. Nevertheless, the strategy showed promise in eliciting neutralizing epitope-specific responses. Further development, including use of the dendrimers as boosting immunogens, may achieve the desired functional activity.

Virus-vectored vaccines are also commonly used to deliver vaccine antigen. They activate DCs effectively, stimulating the innate immune system by their adjuvant capability and triggering stronger adaptive immune responses. Recent studies are exploiting transcriptome analysis to compare DC activation and maturation by various viral vectors. A recent study using pathway and network-oriented genome-wide association analysis (PANOGA) revealed increased activation of the Janus kinase/signal transducers, and activators of transcription (JAK/STAT) pathway correlated with viral vectors that elicited expression of DC co-stimulatory molecules and antigen-specific T cell responses, whereas a significant downregulation of the oxidative phosphorylation pathway, associated with stronger T cell-specific immune responses, was observed only by viral vectors that resulted in robust DC stimulation [57]. As virus-vectored vaccines stimulate DCs directly by inducing various signaling pathways, leading to enhanced antigen-specific immune responses, further analyses of this type will aid selection of vectors for vaccine development. Numerous vectors have been evaluated in designing HIV vaccines, including replicating and nonreplicating adenovirus type 5 (Ad5) and alternative-serotype Ad, poxviruses including vaccinia virus, modified vaccinia Ankara (MVA), and fowlpox virus, lymphocytic choriomeningitis virus (LCMV), cytomegalovirus (CMV), and vesicular stomatitis virus (VSV) [58]. An HIV-1-derived lentiviral vector has also been used to specifically deliver vaccine immunogens to DCs [59]. The various vectors exhibit different cell tropism and immune stimulating properties. Here, we summarize a few recent reports.

It is believed that mucosal immune responses will be required to provide effective protection against HIV infection, as the virus is primarily transmitted across intestinal/genital mucosa. Ad5 infects cells at mucosal inductive sites and therefore has been investigated extensively for HIV vaccine development. Ad5-based HIV vaccines elicit antigen-specific gut-homing CD8 T cell responses shown to be associated with Ad5-induced retinal dehydrogenase (RALDH) enzyme expression in cDCs [60]. RALDH expression can convert vitamin A to retinoic acid, which facilitates the gut-homing potential of T and B cells [61,62]. Replicating Ad5 host-range mutant (Ad5hr) vectored vaccines expressing HIV or SIV antigens have been studied extensively in non-human primates. In a rhesus macaque monkey model, an Ad5hr-GFP recombinant administrated by sublingual, intranasal, intratracheal, vaginal, or rectal routes was shown to express the inserted gene in mDCs up to 25 weeks after the last Ad immunization. This result suggested that there was no restriction in mucosal vaccination route for viral replication and elicitation of immune responses [63]. A more recent study found that, following mucosal immunization by intranasal/oral and intratracheal routes, Ad5hr-SIV recombinants enhanced recruitment, activation, and cytokine production of both mDCs and pDCs at the rectal mucosa within three days after immunizations. The rectal mDCs and pDCs were able to induce antigen-specific CD4 and CD8 T cell proliferation and cytokine production in an in vitro co-culture system. Therefore, the replicating Ad5hr-SIV recombinant vaccine could elicit antigen-specific immune responses in mucosal tissue by enhancing DC recruitment, stimulation, migration to lymph nodes, and antigen presentation [64]. A caveat must be pointed out regarding attempts to elicit mucosal immunity, however. In the clinical trial of an Ad5-HIVgag vaccine (the STEP trial), HIV acquisition was increased in uncircumcised individuals with high pre-existing Ad5 antibody titers [65]. It has been shown that Ad5 immune complexes can activate the DC–T cell axis and induce expansion of mucosal-homing memory CD4 T cells, thus providing more target cells for HIV infection [66,67]. Whether this mechanism was responsible for the STEP study failure is not known, but care must be taken with all vaccine strategies to ensure that potential mucosal target cells are appropriately balanced by additional immune protective responses.

Poxviruses are among the most heavily used viral vectors for HIV vaccines and have also been shown to interact with DCs. Various vectors elicit different outcomes, however, highlighting the need to carefully examine mechanisms involved in desired immune responses. For example, recruitment of conventional DCs to the lung mucosa and elicitation of CD8 T cells with higher avidity following intranasal immunization of mice with recombinant HIV-fowlpox virus could be enhanced by transient inhibition of IL-4/IL-13 at the site of vaccination. In contrast, immunization with HIV-vaccinia virus or MVA virus vaccines increased a different subset of lung-derived DCs with lessened avidity [68]. The route and the method of immunization with poxvirus vectors can also influence induced responses. Sublingual/buccal needle-free vaccinations of HIV-expressing MVA vector and gp120 proteins generated strong mucosal and systemic IgG responses as well as CD4 and CD8 T cell responses in rhesus macaques. Better protective efficacy against SHIV infection was observed compared to topical application of the vaccine to the same site. The authors demonstrated that DCs in the buccal and the sublingual tissue increased RALDH activity upon activation and transferred vaccine antigens to lymph nodes to improve vaccine efficacy [69]. The needle-free vaccination method thus provides an alternative to standard sub-cutaneous or intradermal infection.

Among other viral vectors where interaction with DCs has been explored, recombinant Newcastle disease virus (rNDV) vector expressing HIV Gag showed type 1 IFN induction and DC maturation without any safety issues [70]. Subsequently, in addition to rNDV-Gag, a single-chain Fv antibody specific for the DC-restricted antigen uptake receptor DEC205 was added to the vaccine regimen. This new strategy exhibited enhanced Gag-specific CD4 and CD8 T cell activities and better protection against Gag-expressing vaccinia virus infection due to increased DC-targeted immune activation [71].

The HIV-1 lentiviral vector vaccine has delivered antigenic proteins to DCs by pseudotyping the vector with a mutant Sindbis virus glycoprotein, which allows binding to DC-SIGN on DCs, vector transduction, and stimulation of antigen-specific immune responses [59]. Activation of DCs by lentiviral vectors was shown to be induced by the host STING and the cGAS pathway rather than by signaling of TLR or retinoic acid-inducible gene-1 (RIG-1)-like receptors. Activation by myeloid differentiation primary response 88 (MyD88), toll/IL-1 receptor (TIR)-domain-containing adapter-inducing interferon-β (TRIF), and MAVS signaling pathways was not involved [72].

## 4. Roles of DCs in Therapeutic HIV Vaccines

To control HIV infection, combination antiretroviral therapy (cART) has effectively treated HIV-infected patients, leading to undetectable viral loads. However, cART only inhibits viral replication and associated disease progression rather than completely eliminating integrated virus. Moreover, long-term cART has adverse health effects and leads to development of viral resistance as well as economic burdens. Therefore, new effective therapeutic strategies need to be developed. For this purpose, many different types of therapeutic HIV vaccines that aim to restore HIV-specific T cell responses and control HIV replication without cART have been evaluated. It has long been expected that DCs would play roles in these therapeutic vaccine mechanisms inducing antigen-specific immune responses and restoring CD4 T cell activity against HIV [73,74,75]. It is also known that DCs can act as natural latency reversing agents following their maturation and expression of TNF-α [76]. Thus, DCs may play a key role in therapeutic vaccine approaches by reactivating HIV, allowing expression of viral antigens on infected cells, and providing targets for cytotoxic CD8 T cells. In fact, a recent study reported that antigen presentation by MoDCs from chronically HIV-infected individuals to autologous CD4 T cells resulted in latency reversal dependent on the CD40-CD40L pathway and also was able to activate expansion of autologous antigen-specific CD8 cytotoxic T cells [77]. Here, we summarize several therapeutic vaccine approaches and highlight those where the roles of DCs have been elucidated.

Whole inactivated virus, recombinant protein, and DNA vectors have been tested as therapeutic vaccine antigens and have generally been found to be poorly immunogenic, eliciting weak antigen-specific CD4 T cell and antibody responses, as well as limited in controlling viral replication. This poor performance might be due to the impaired immune system of HIV-infected individuals, including the loss of DCs in chronic infection [78]. Therefore, a number of studies have reverted to use of autologous DCs pulsed with various HIV antigens. Moreover, recent therapeutic vaccines have aimed to induce broad immune responses rather than single antigen-specific responses to combat viral escape mutants and suppress the viral rebound [79]. For example, ALVAC-HIV vaccine expressing portions of HIV env, gag, pol, and nef genes, and Lipo-6T (tetanus toxoid class II-restricted universal CD4 epitope combined with 2 Gag, 2 Nef, and 1 Pol peptide) immunization followed by IL-2 enhanced CD4 and CD8 T cell responses, improved viral control and reversed CD4 exhaustion [80,81,82]. The peptides in the Lipo-6T vaccine component were modified by addition of a lipid tail in the C-terminal region to improve antigen presentation. Similarly, immunization of HIV^+^ patients stable on cART with autologous DCs electroporated with mRNA encoding Tat, Rev, and Nef enhanced vaccine-specific CD4 and CD8 T cell responses. However, following treatment interruption, there was no correlation seen between the induced responses and the subsequent weeks off treatment [83]. A follow-up study using analysis of plasma analytes identified thyroxine-binding globulin as a potential biomarker of DC vaccination efficacy, as it was associated with a longer time off ART, as were neutrophil counts [84]. A recent therapeutic trial immunized cART-treated HIV^+^ patients with autologous DC enriched by ex vivo culture with GM-CSF and IFN-α and loaded with LIPO5 (2 Nef, 2 Gag, and 1 Pol lipopeptides). Immune responses targeted both dominant and sub-dominant epitopes in the five immunogens [85]. In spite of these promising findings, careful HIV antigen selection for DC stimulation is needed, because some HIV proteins impair DC functions. Nef and Vpr proteins of HIV have been shown to alter DC maturation and T cell activation by regulation of co-stimulatory molecules and cytokine production [86,87,88]. It may be that specific peptides, as opposed to intact proteins, do not mediate such inhibitory functions, allowing them to serve as appropriate immunogens.

Aside from combination strategies with other vaccine components, DCs alone have been applied as cellular adjuvants based on the rationale that, since general functions of DCs such as cytokine production and antigen presentation are impaired by HIV infection, use of autologous DCs stimulated by HIV antigens ex vivo will elicit protective immune responses in HIV infected patients. MoDCs are generated ex vivo from monocytes of HIV infected patients by GM-CSF and IL-4 treatment [89]. Even though the cells are from HIV-infected individuals, these ex vivo generated MoDCs are mostly uninfected and functionally intact [90,91]. The healthy MoDCs are then stimulated with HIV antigens such as whole inactivated HIV virus, recombinant envelope glycoproteins, peptides, or recombinant viral vectors to load HIV viral peptides on their surfaces. This vaccine strategy has been considered safe and effective because autologous DCs are used. Additionally, different types of antigens can be loaded, and ex vivo cell manipulation and treatment are somewhat easier and more predictable than in vivo DC-targeted therapeutic vaccinations. The HIV antigen-loaded DCs produced cytokine and chemokines and elicited CD4 and CD8 T cell proliferation and CD8 T cell-mediated killing, leading to decreased viral loads. The HIV-loaded autologous DC vaccines could enhance HIV-specific antibody responses as well [92,93,94]. For example, aldrithiol (AT)-2-inactivated SIVmac251-loaded autologous MoDCs or unloaded autologous MoDCs were administered to SIV-infected rhesus macaques three times at two week intervals. Animals that received antigen-loaded MoDCs exhibited 50-fold and 1000-fold decreases of SIV DNA and RNA in blood, respectively. The viral control was maintained more than 34 weeks. In addition, both humoral and cellular SIV-specific immune responses were induced [95]. These results suggested that antigen-loaded DCs could be a promising therapeutic vaccine strategy to induce anti-viral responses and antigen-specific protective immune responses in HIV-infected individuals. Subsequently, many phase I clinical trials were evaluated to confirm the safety of DC inoculations, and phase II trials have investigated the therapeutic efficacy of the DC vaccines in HIV-1 infected patients [89,96,97,98].

As with prophylactic vaccines, nanotechnology has facilitated development of DC-targeted vaccines. Following antigen acquisition, DCs require activation and maturation by an adjuvant in order to effectively mediate immune responses. Nanoparticles can encapsulate both antigen and adjuvant, thus ensuring delivery of both [35]. Recently, mRNA HIV vaccines are being investigated, as they can encode both multiple antigens and activation signals yet are safe, efficacious, and easily produced (for review, see [99]). A difficulty with these vaccines, however, involves development of methods to easily deliver them to DCs. For example, an mRNA vaccine encoding HIV T cell immunogen sequence from Gag, Pol, Vif, and Nef together with three activation signals activated DCs, upregulated maturation markers, and elicited cytokine production and enhanced T-cell proliferation along with CTL responses [100]. Administration to DCs, however, was by ex vivo electroporation of MoDCs from HIV-infected patients or intranodal immunizations of mice. Fortunately, new methods of delivery involving nanoparticles are being developed. A recent study made use of polylactic acid nanoparticles and polyplexes composed of mRNA encoding HIV Gag and cationic cell-penetrating peptides. The complexes were shown to be readily taken up by DCs in vitro by phagocytosis and clathrin-dependent endocytosis, leading to protein expression and activation [101]. Other methods such as the use of dendrimers, as discussed above (and [56]), have facilitated vaccine delivery to DCs in vivo. Dendrimers are branched nanopolymers that can have various functional groups on the terminal branches, allowing conjugation of multiple vaccine and activating components and targeting ligands to a single dendrimer [102]. The targeting molecules can facilitate delivery to DCs in vivo. The rapidly expanding field of nanobiology should lead to improved immunotherapeutic vaccines with greater breadth, potency, and ease of application.

## 5. Closing Remarks

The goals of prophylactic and therapeutic vaccine development are quite similar: induction of potent and broad immune responses specific for HIV antigens and able to resist viral escape mutants. The arsenals available to generate such responses are also similar for both approaches and include DCs themselves as critical mediators of innate and adaptive immunity as well as protein, nucleic acid, and viral vectored vaccines. The approaches differ in some respects, however, not the least of which is the issue of latency in HIV infected individuals and the need to stimulate resting memory cells residing anywhere in the body to express HIV antigens susceptible to viral specific CD8 T cells and functional antibodies able to kill or eliminate infected cells. On the other hand, preventive vaccines may initially focus on mucosal sites of transmission and elicitation of antibodies with neutralizing activity. As described in this brief review, several new approaches should help DCs mediate the necessary immune responses and facilitate development of vaccine strategies appropriate for both types of vaccine. The first is continued development of mRNA vaccines that can incorporate not only a spectrum of viral antigens but also necessary adjuvants for optimal DC presentation and maturation. Second, an increasing variety of nanoparticles that can be directed in vivo to specific DC targets provides a mechanism to target specific mucosal and systemic tissues and induce potent immune responses. Finally, the increasing use of transcriptome analysis to decipher mechanisms of action of specific DC subsets, viral vectors, and adjuvants should allow greater accuracy in selection of vaccine components for specific functions. In addition, the use of such “omics” approaches should facilitate monitoring of effective DC-based vaccine strategies. We expect that continued progress in all these areas will result in more rapid movement towards the efficacy desired in both prophylactic and therapeutic vaccines.
